# Community based interventions for the prevention and control of tuberculosis

**DOI:** 10.1186/2049-9957-3-27

**Published:** 2014-08-01

**Authors:** Ahmed Arshad, Rehana A Salam, Zohra S Lassi, Jai K Das, Imama Naqvi, Zulfiqar A Bhutta

**Affiliations:** 1 Division of Women and Child Health, The Aga Khan University, 74800 Karachi, Pakistan; 2 Center of Excellence in Women & Child Health, The Aga Khan University, Karachi, Pakistan; 3 Center for Global Child Health Hospital for Sick Children, Toronto, Canada

**Keywords:** Community-based interventions, Tuberculosis, DOTS, integrated delivery, CHWs

## Abstract

In 2012, an estimated 8.6 million people developed tuberculosis (TB) and 1.3 million died from the disease. With its recent resurgence with the human immunodeficiency virus (HIV); TB prevention and management has become further challenging. We systematically evaluated the effectiveness of community based interventions (CBI) for the prevention and treatment of TB and a total of 41 studies were identified for inclusion. Findings suggest that CBI for TB prevention and case detection showed significant increase in TB detection rates (RR: 3.1, 95% CI: 2.92, 3.28) with non-significant impact on TB incidence. CBI for treating patients with active TB showed an overall improvement in treatment success rates (RR: 1.09, 95% CI: 1.07, 1.11) and evidence from a single study suggests significant reduction in relapse rate (RR: 0.26, 95% CI: 0.18, 0.39). The results were consistent for various study design and delivery mechanism. Qualitative synthesis suggests that community based TB treatment delivery through community health workers (CHW) not only improved access and service utilization but also contributed to capacity building and improving the routine TB recording and reporting systems. CBI coupled with the DOTS strategy seem to be an effective approach, however there is a need to evaluate various community-based integrated delivery models for relative effectiveness.

## Multilingual abstracts

Please see Additional file 1 for translations of the abstract into the six official working languages of the United Nations.

## Introduction

Tuberculosis (TB) remains a major global health problem. In 2012, an estimated 8.6 million people developed TB and 1.3 million died from the disease [1]. The number of TB deaths is unacceptably large given that most of these deaths are preventable. With the recent resurgence related to the human immunodeficiency virus (HIV), TB prevention and management has become further challenging [2-4]. TB is preventable as well as curable and its transmission could be prevented by prompt identification and treatment of the infected person. However; ensuring treatment completion is crucial for the prevention of relapse and secondary drug resistance. The World Health Organization (WHO) recommends Stop TB Strategy based on the Directly Observed Therapy, Short-course (DOTS) to control TB. The strategy aims to ensure that patients take a standard short-course of chemotherapy under guided supervision to cure the disease as well as to prevent transmission. Patients are assisted through their treatment regimen and encouraged to treatment completion in order to prevent resistance to the available anti-TB drugs. DOTS has been delivered by health workers, community volunteers, lay health workers and even family members [5]. For further details on TB burden, epidemiology and intervention coverage, refer to previous paper in this series [6].

Considering the recent shift in epidemiological presence of TB, there is a legitimate call for integration of therapeutic services especially with HIV [7]. Since both diseases require long term treatment regimens, community based support may play a defining role towards prevention and control of these syndemic diseases of poverty. Moreover, integration of services in low-income countries may prove beneficial in terms of cost-effectiveness and decrease demand on health service infrastructure. However, there is a need to gauge whether these strategies lead to effective treatment outcomes. This paper aims to evaluate the effectiveness of community based interventions (CBI) for the prevention and treatment of TB.

## Methods

We systematically reviewed literature published by September 2013 to identify studies evaluating CBI for TB as outlined in our conceptual framework [8]. Our priority was to select existing randomized controlled trials (RCT), quasi-experimental and before/after studies in which the intervention was delivered within community settings and the reported outcomes were relevant. A comprehensive search strategy was developed using appropriate key words, Medical Subject Headings (MeSH) and free text terms. The search was conducted in PubMed, Cochrane libraries, EMBASE and WHO Regional Databases; additional studies were identified by hand searching references from included studies. Studies were excluded if the intervention was purely facility-based or had a facility-based component. Studies that met the inclusion criteria were selected and double data abstracted on a standardized abstraction sheet. Quality assessment of the included RCT was done using the Cochrane risk of bias assessment tool [9]. We conducted meta-analysis for individual studies using the software Review Manager 5.1. Pooled statistics were reported as the relative risk (RR) for categorical variables and standard mean difference (SMD) for continuous variables between the experimental and control groups with 95% confidence intervals (CI). Subgroup analysis was conducted for therapeutic and preventive (screening) CBI, integrated and non-integrated CBI and by type of studies. The detailed methodology is described in previous paper [8].

## Review

**Figure 1:**
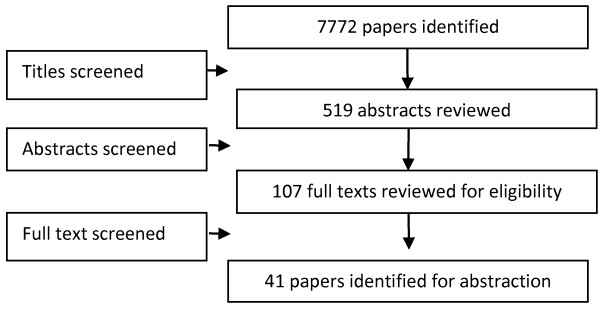
Search flow diagram.

A total of 7,772 titles were identified from all databases and 107 full texts were screened. After screening, forty one [10-50] studies met the inclusion criteria; 34 RCT and 7 before/after studies (Figure 1). From the included RCT, 18 were adequately randomized while five studies reported adequate sequence generation (Table 1). Due to the nature of the intervention, blinding of the participants and assessors was not possible. Studies provided insufficient information on selective reporting which limited us from making any judgment. Ten of the included studies focused on TB prevention and case detection while 31 studies were on treatment of patients with active TB. Interventions involved community based delivery of DOTS; community mobilization and support; education and training; and monetary incentives for treatment adherence. Most of the CBI utilized community health workers (CHW) or family members as part of the delivery strategy. Table 2 describes the characteristics of included studies.

**Table 1:** Quality assessment of the included RCTs

**Article**	**Randomization**	**Sequence generation**	**Allocation concealment**	**Blinding of participants**	**Blinding of assessors**	**Selective reporting**
Atkins 2011 [11]	No	No	No	Not clear	Not clear	Not clear
Barker 2002 [12]	No	No	Not clear	Not clear	Not clear	No
Clarke 2005 [15]	Yes	No	Yes	No	No	No
Colvin 2003 [16]	No	No	No	Not clear	Not clear	Yes
Corbett 2010 [17]	Yes	Not clear	Yes	No	Yes	No
Dudley 2003 [19]	No	No	No	Not clear	Not clear	No
Fairall 2005 [20]	Yes	Yes	Not clear	No	Yes	No
Ferreira 2011 [21]	No	No	No	Not clear	Not clear	No
Filho 2009 [14]	No	No	No	Not clear	Not clear	Yes
Kironde 2002 [23]	No	No	No	Not clear	Not clear	No
Vieira 2011 [31]	No	No	No	Not clear	Not clear	Not clear
White 2002 [33]	Yes	Yes	Yes	Not clear	Not clear	No
Zwarenstein 2000 [35]	Yes	Yes	Yes	No	No	No
Vassal 2002 [30]	Not clear	Not clear	Not clear	Not clear	Not clear	Not clear
Prado 2011 [27]	No	No	No	No	No	No
Mafigiri 2012 [24]	No	No	No	No	Not clear	No
Niazi 2003 [26]	No	No	No	No	Not clear	No
Uwimana 2012 [28]	Yes	Not clear	Not clear	Not clear	Not clear	No
Yassin 2013 [34]	No	No	No	Not clear	Not clear	No
CDI group	Yes	Not clear	Not clear	Not clear	Not clear	Not clear
Miti 2003 [25]	No	No	No	Not clear	Not clear	No
Zwarenstein 1998 [50]	Yes	Not clear	Not clear	No	No	No
Chaisson 2001 [37]	Yes	Yes	Yes	Not clear	Not clear	No
Heal 1998 [39]	No	No	No	No	No	No
Kamolratanakul 1999 [40]	Yes	Yes	Yes	Yes	No	No
Khan 2002 [41]	Yes	Not clear	Not clear	Not clear	Not clear	No
Lwilla 2003 [42]	Yes	Not clear	Not clear	No	No	Yes
MacIntyre 2003 [43]	No	No	No	No	Yes	Yes
Malotte 2001 [44]	Yes	No	Yes	Not clear	Not clear	No
Newell 2006 [45]	Yes	No	Yes	No	Yes	No
Ollé-Goig 2001 [46]	No	No	No	No	No	No
Walley 2001 [47]	Yes	Yes	Yes	No	Yes	No
Wandwalo 2004 [48]	Yes	No	No	No	No	No
Wright 2004 [49]	Yes	No	Yes	Not clear	Not clear	No

**Table 2:** Characteristics of the included studies

**Study**	**Study design**	**Country**	**Intervention**	**Target Population**	**Integrated/Non-Integrated**
Gandhi 2008 [38]	Before/after	South Africa	DOTS therapy integrated with anti-retroviral therapy on a community level	TB and HIV co-infected adults	Integrated
Chaisson 2001 [37]	RCT	USA	Preventive Isoniazid therapy for injection drug users	Injection drug users	Non-integrated
Zwarenstein 1998 [50]	RCT	South Africa	Self-supervised treatment compared against therapy observed in clinic	Adult pulmonary TB patients	Non-integrated
Heal 1998 [39]	Quasi-trial	Canada	Self-administered preventive therapy compared against preventive therapy observed in a clinic	All aboriginals in British Columbia undergoing preventive therapy for TB	Non-integrated
Kamolratanakul 1999 [40]	RCT	Thailand	DOTS therapy compared against self-supervised therapy	All smear positive pulmonary TB patients	Non-integrated
Khan 2002 [41]	RCT	Pakistan	DOTS therapy compared against self-supervised therapy	Adults with TB	Non-integrated
Lwilla 2003 [42]	RCT	Tanzania	Community-based DOTS compared against institution-based DOTS	All patients diagnosed with TB at selected health centres	Non-integrated
MacIntyre 2003 [43]	Quasi-trial	Australia	Family member supervised DOTS compared against non-observed therapy	All patients diagnosed with TB at selected health centres	Non-integrated
Malotte 2001 [44]	RCT	USA	INH therapy of latent TB infections given either by outreach workers or at a facility	People with active or recent history of drug use	Non-integrated
Newell 2006 [45]	RCT	Nepal	Comparison between community-members DOTS and family member DOTS	All new smear positive cases of pulmonary TB	Non-integrated
Ollé-Goig 2001 [46]	Quasi-trial	Haiti	DOTS compared with non-observed therapy	Adult TB patients	Non-integrated
Walley 2001 [47]	RCT	Pakistan	DOTS by family member compared with DOTS by healthcare worker and non-observed therapy	Adult TB patients	Non-integrated
Wandwalo 2004 [48]	RCT	Tanzania	Community-based DOTS compared against healthcare worker DOTS	TB patients of all ages	Non-integrated
Wright 2004 [49]	RCT	Swaziland	Community health worker DOTS compared with family member DOTS	TB patients of all ages	Non-integrated
Atkins 2011 [11]	Quasi-trial	South Africa	Enhanced tuberculosis treatment adherence	Adult TB patients	Integrated
Barker 2002 [12]	Quasi-trial	South Africa	Community-based DOTS compared against healthcare worker DOTS	TB patients of all ages	Non-integrated
Clarke 2005 [15]	RCT	South Africa	Comparison between conventional TB treatment and lay health worker DOTS	Adult TB patients	Non-integrated
Colvin 2003 [16]	Quasi-trial	South Africa	Traditional healers mobilized as DOTS supervisors	TB patients of all ages	Non-integrated
Corbett 2010 [17]	RCT	Zimbabwe	Door-to-door and mobile van announcements compared as strategies to increase TB detection	All people in a specific community	Integrated
Diez 1996 [18]	Before/after	Spain	Social support for deserving TB patients	Adult TB patients	Non-integrated
Dudley 2003 [19]	Quasi-trial	South Africa	DOTS compared with non-observed therapy	Adult TB patients	Non-integrated
Fairall 2005 [20]	RCT	South Africa	Educational outreach to nurses to increase TB case detection	Patients attending specific clinics	Integrated
Ferreira 2011 [21]	Quasi-trial	Brazil	DOTS compared with non-observed therapy	TB patients of all ages	Non-integrated
Filho 2009 [14]	Quasi-trial	Brazil	Food baskets offered to patient to assess effect on treatment outcomes	Adult TB patients	Integrated
Kamineni 2011 [22]	Before/after	India	Increasing case detection and treatment adherence, decreasing stigma and discrimination, empowering affected people, and mobilising political commitment and resources	TB patients of all ages	Non-integrated
Kirondea 2002 [23]	Quasi-trial	South Africa	Assessing the feasibility of using lay volunteers as DOTS supervisors	Adult TB patients	Non-integrated
Vieira 2011 [31]	Quasi-trial	Brazil	DOTS compared with non-observed therapy	Adult TB patients	Non-integrated
Weis 1994 [32]	Before/after	USA	DOTS compared with non-observed therapy	TB patients of all ages	Non-integrated
White 2002 [33]	RCT	USA	Incentivized treatment compared with no incentive	Susceptible population in a county jail	Non-integrated
Zwarenstein 2000 [35]	RCT	South Africa	DOTS compared with non-observed therapy	TB patients of all ages	Non-integrated
Prado 2011 [27]	Quasi-trial	Brazil	Community health worker DOTS compared with family member DOTS	Adult TB patients	Non-integrated
Vassall 2002 [30]	Quasi-trial	Syria and Egypt	Community-based DOTS compared against institution-based DOTS	TB patients of all ages	Integrated
CDI study group	RCT	Nigeria, Uganda and Cameroon	Integration of community interventions to counter multiple diseases through a single framework	TB patients of all ages	Integrated
Miti 2003 [25]	Quasi-trial	Zambia	Integration of HIV and TB services	Adult TB patients	Integrated
Amo-Adjei 2013 [10]	Before/after	Ghana	Improvements in diagnosis, community TB care and stigma reduction among community and health workers towards TB patients	Adult TB patients	Integrated
Brust 2012 [13]	Before/after	South Africa	Integration of HIV and TB services	Adult TB patients	Integrated
Mafigiri 2012 [24]	Quasi-trial	Uganda	Community-based DOTS compared against institution-based DOTS	TB patients of all ages	Non-integrated
Niazi 2003 [26]	Quasi-trial	Iraq	Community-based DOTS compared against institution-based DOTS	Adult TB patients	Non-integrated
Uwimana 2012 [28]	RCT	South Africa	Training community care workers (CCWs) to provide integrated care	All members of localities where the CCWs were based	Integrated
Uwimana 2013 [29]	Before/after	South Africa	Training community care workers (CCWs) to provide integrated care	All members of localities where the CCWs were based	Integrated
Yassin 2013 [34]	Quasi-trial	Ethiopia	Training, engaging stakeholders and communities and active case-finding by female Health Extension Workers (HEWs) at village level	All members of localities where the HEWs were based	Non-integrated

### Quantitative synthesis

**Figure 2:**
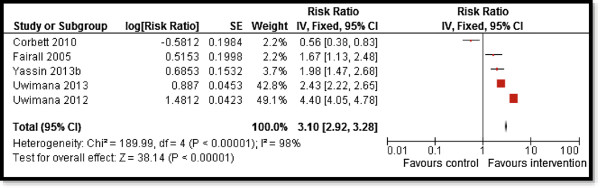
Forest plot for the impact of CBI on TB case detection.

**Figure 3:**
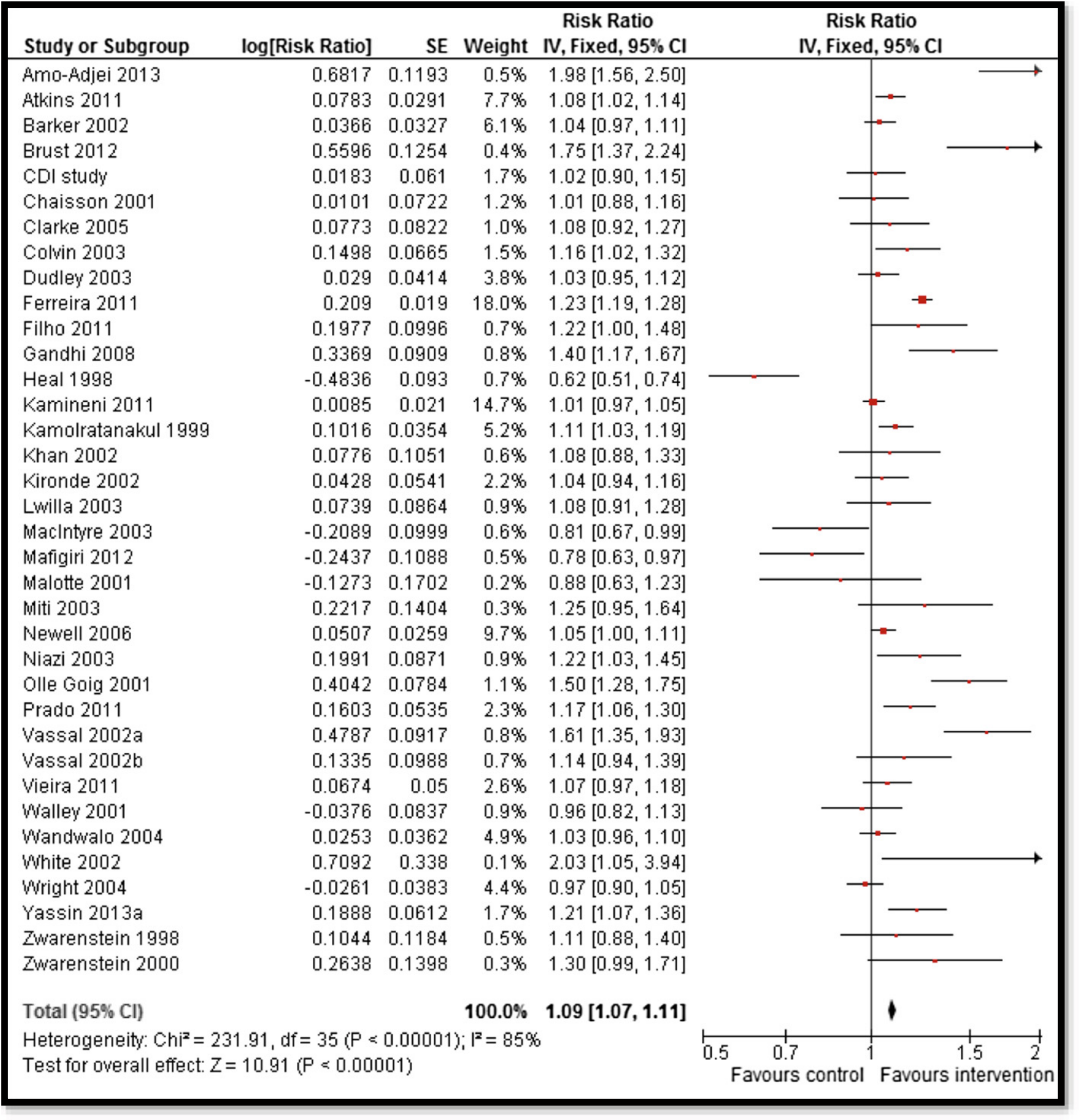
Forest plot for the impact of CBI on treatment success rate.

Overall, CBI for TB prevention and case detection showed significant increase in TB detection rates (RR: 3.1, 95% CI: 2.92, 3.28) (Figure 2) while there was a non-significant impact on TB incidence, although this evidence is from a single study. Subgroup analysis showed consistent results for various study designs and whether the interventions were delivered in an integrated or a non-integrated manner. CBI for treating patients with active TB showed an overall improvement in treatment success rates (RR: 1.09, 95% CI: 1.07, 1.11) (Figure 3) and evidence from a single study suggests significant reduction in relapse rate (RR 0.26, 95% CI: 0.18, 0.39). The results were consistent for various study design and delivery mechanism. The results are summarized in Table 3.

**Table 3:** Results for overall and sub-group analysis according to type of study, intervention and treatment

**Outcomes**	**Estimates (95% CI)**					
	**Prevention/Screening**	**Therapeutic management**	**RCT/Quasi**	**Pre-post studies**	**Integrated**	**Non-integrated**
**CBI for TB case detection**						
**TB detection**	*3.10 [2.92, 3.28]*	-	*3.71 [3.44, 4.01]*	*2.43 [2.22, 2.65]*	*3.15 [2.97, 3.34]*	*1.98 [1.47, 2.68]*
	5 datasets, 5 studies		4 datasets, 4 studies	1 dataset, 1 study	4 datasets, 4 studies	1 dataset, 1 study
**TB incidence**	0.63 [0.36, 1.09]	-	-	0.63 [0.36, 1.09]	-	0.63 [0.36, 1.09]
	1 dataset, 1 study			1 dataset, 1 study		1 dataset, 1 study
**CBI for patients with Active TB**						
**Treatment success**	-	*1.09 [1.07, 1.11]*	*1.10 [1.08, 1.12]*	*1.06 [1.02, 1.10]*	*1.29 [1.21, 1.38]*	*1.08 [1.06, 1.10]*
		36 datasets 35 studies	32 datasets, 31 studies	4 datasets, 4 studies	8 datasets, 7 studies	28 datasets, 28 studies
**Relapse**	-	*0.26 [0.18, 0.39]*	-	*0.26 [0.18, 0.39]*	**-**	*0.26 [0.18, 0.39]*
		1 dataset, 1 study		1 dataset, 1 study		1 dataset, 1 study

Italics denote statistically significant estimates.

### Qualitative synthesis

Included studies suggest that CBI for TB have the potential to improve access to diagnostic and treatment services for poor rural communities and vulnerable population including women and children. Community based TB treatment delivery through CHW not only improved access and service utilization but also contributed to capacity building and improving routine TB recording and reporting systems through regular supportive supervision [34]. Better outcomes were reported when DOTS was provided together with CHW program as it enabled treatment continuation; thus achieving higher treatment success rates [21]. Studies also support the feasibility of integrating cadres of CHW through establishing supportive structures and supervision [28,29]. Besides treatment, community based untargeted periodic active case finding for symptomatic smear-positive TB also made a substantial contribution to diagnosis and control of infectious TB [17]. This is a significant finding as the slow rate at which patients with tuberculosis report to health facilities is a major limitation in global efforts to control TB. However, especial emphasis needs to be given for training, close supervision and support for CHW to achieve job satisfaction and sustainability [34].

Despite considerable advocacy for increased collaboration and integration of TB and HIV care, few models of integration have been implemented, evaluated and reported [11,17,20,28,29]. However, existing evidence favors integrating TB and HIV care for improving active case finding and early diagnosis of TB, which in turn, reduces the risk of TB transmission [28]. TB/HIV co-infected patients receiving concurrent antiretroviral and TB therapy achieved high levels of adherence and excellent TB and HIV outcomes [28]. Integrated provision of TB, HIV and prevention of mother to child transmission (PMTCT) services at community level through CHW is feasible, acceptable and successful [28,29]. Training CHW to provide a comprehensive package of TB ⁄HIV⁄PMTCT prevention, case finding and treatment support services can bridge the current gaps in service delivery through vertical TB, HIV and PMTCT programs. Evidence also suggests that DOTS strategy can be successfully implemented at primary health care clinics [31]. However such integrations should follow careful planning and caution with greater investments in developing and implementing infection control and laboratory infrastructure [28].

Key components reported for a successful community delivery strategy to prevent and treat TB included a preexisting TB DOTS infrastructure, patient treatment literacy training, and adherence support from trained CHW and family members [21]. Involvement of non-governmental organization (NGO) has also been reported as an essential component of TB programs [22]. Formation of community groups also have reported to contribute towards improved awareness and knowledge about TB and treatment adherence. Community groups help bridge gaps between health system and community through support and coordination. Multi-sectoral community mobilization events that engage community leaders is also one of the enabling tools for successful community based programs in TB⁄HIV⁄PMTCT care [28,29]. Engagement of successfully treated patients can also assist in reducing community stigma and discrimination [22]. Other strategies for organizing, coordinating and managing health care include continuous education and direct supervision of health providers; establishment of goals and regular monitoring of process and result indicators; and incentives for effective use of recommended guidelines [21].

One of the major reported barriers in the success of TB programs is non-adherence mainly due to the lack of support. The intensity of support for patients is reported to diminish in the continuation phase of treatment [11]. Lack of incentives, difficult treatment access, poor communication between health providers and patients, poor application of DOTS, lack of active search for missing patients, and limitations of supervision in treatment units are recognized barriers to treatment success [21]. In addition, the presence of multiple cadres of CHW providing TB and HIV services in silos has hindered the enhancement of collaborative TB⁄HIV activities in community, as well as their supervision [28,29]. Inconsistency in the supply of commodities such as test kits need to be resolved to increase uptake of HIV testing and counseling [28,29].

## Discussion

Our review findings suggest that CBI are effective in TB detection and treatment but its role in preventing TB cases has not been comprehensively evaluated. Community based delivery of DOTS may be more feasible and effective for TB case detection and treatment as community workers are familiar with the layout of community and have community member’s trust which healthcare officials would have to develop. Moreover, a community-based approach helps empower each community to deal with its own problems and also provides patient with a greater degree of autonomy and satisfaction with the treatment regime [51]. This involvement of respected, responsible and resourceful community and family members increases the trust that is required to initiate treatment and provides close supervision thereby maximizing adherence which is crucial in such a lengthy treatment regime. Limited coverage of public health services has continued to impede accelerated access to TB control services due to inadequate health service infrastructure, insufficient decentralization of services and inadequate human, material and financial resources. Hence, community delivery platforms offer improved access and equitable distribution of care.

High incidence of TB and its significant financial burden makes it imperative to find a plausible strategy to cope with this disease. The fact that it effects lower socio-economic groups further compounds the problem. Gender inequality, social stigma, and poverty are also recognized as important barriers for successful TB prevention and control programs [52-55]. In light of the above situation, DOTS provides a successful and cost-effective strategy to deal with the burden of TB [27,30,41]. CBI coupled with DOTS seems to be an effective approach as they have the potential to maximize the outreach and minimize the cost. Community based TB control also offers many lessons for the control of HIV epidemic. With the emergence of HIV and consequent TB resurgence, a comprehensive and equitable strategy is needed to stem the worsening double burden of these two infections in poor countries [56].

The WHO currently advocates home-based care and integrated management of dually infected TB/AIDS patients [57]. It recommends a 12 point package of collaborative TB/HIV activities based on creating a mechanism of collaboration between TB and HIV programs, reducing the burden of TB among people living with HIV and reducing the burden of HIV among TB patients. CHW delivering DOTS can be further trained to carry out this additional task and studies are needed to evaluate the feasibility, relative effectiveness and cost effectiveness of this approach [23,58]. However, such integration would involve CHW training and time; improved collaboration between community and facility; and strengthening referral services [59,60].

## Conclusion

Well-designed operational research is needed to pragmatically evaluate various models of community based delivery. There is a need to evaluate and address context specific barriers to community based implementation, especially for collaborative TB⁄HIV activities in the community to avoid duplication of labor and resources. Future studies should focus on evaluating novel community delivery models for their success in larger and more diverse populations and impact TB prevention and active case detection.

## Abbreviations

CBI: Community based interventions; CHW: Community health workers; CI: Confidence interval; DOTS: Direct observed therapy; HIV: Human immunodeficiency virus; MDR-TB: Multi drug resistant tuberculosis; NGO: Non-governmental organization; PMTCT: Prevention of mother-to-child transmission; RCT: Randomized controlled trial; RR: Relative risk; SMD: Standard mean difference; TB: Tuberculosis; WHO: World Health organization.

## Competing interest

The authors declare that they have no financial or non-financial competing interests.

## Authors’ contribution

ZAB was responsible for designing and coordinating the review. AA and IN were responsible for: data collection, screening the search results, screening retrieved papers against inclusion criteria, appraising quality of papers and abstracting data. RAS, JKD and ZSL were responsible for data interpretation and writing the review. ZAB critically reviewed and modified the manuscript. All authors read and approved the final manuscript.

## Supplementary Material

Additional file 1 Multilingual abstracts in the six official working languages of the United Nations. Click here for file
