# Supplementary material for: Community based interventions for the prevention and control of tuberculosis
Source: Infect Dis Poverty. 2014 Aug 1;3:27. doi: 10.1186/2049-9957-3-27 (PMC4136404; doi:10.1186/2049-9957-3-27)
Supplement: Additional file 1 — Multilingual abstracts in the six official working languages of the United Nations. [file 2049-9957-3-27-S1.pdf]

Translation of the abstract into the six official working languages of the United Nations

التدخلات المجتمعية بهدف الوقاية ومكافحة مرض السلّ

أحمد أرشاد، ريحانة أ سلام، زهرا س لاسّي، إيمانة ناقوي، ذو الفقار أ بوتا.

خلاصة:

سجّل عام 2012، ما يناهز 8.6 مليون إصابة بالسلّ و1.3 وفاةً جرّاء هذا الوباء. هذا وقد شهدنا مؤخراً عودة ظهور هذا المرض المرتبط بفيروس نقص المناعة البشرية، مما يجعل من عملية إدارة والوقاية منه أمراً بالغ الصعوبة. ترمي هذه الورقة الى تقييم فعالية التدخلات المجتمعية في مجال مكافحة والوقاية من السلّ. في هذا السياق، تشير الخلاصات المستمدّة من 41 دراسة حول فعالية التدخلات المجتمعية، أنه غالباً ما لهذا النوع من النشاطات فيما يتعلّق بكشف حالات السلّ والوقاية منه، تأثير ملحوظ على تشخيص هذا الداء (RR: 3.1, 95% CI: 2.92, 3.28)؛ في حين بيّنت دراسة وحيدة تأثيرات خفيفة للتدخلات المجتمعية على الإصابة بالسلّ. هذا وقد أظهرت التدخلات المجتمعية لمعالجة المرضى المصابين بالسلّ النشط أو المعدي، تحسّناً عاماً في معدّلات العلاج (RR: 1.09, 95% CI: 1.07, 1.11) كما دلّت مخرجات دراسة واحدة على انخفاض بارز في معدلات الإنتكاس (RR 0.26, 95% CI: 0.18, 0.39). كما وبدت النتائج منسجمة ومتسقة ومخطط الدراسة وآلية التنفيذ. بدورها أظهرت عملية التحليل النوعي أن علاج السلّ المستند على التدخلات المجتمعية من قبل عاملي الصحة المحليين، قدّ حسّن وبشكل كبير الوصول والإستفادة من الخدمة المذكورة أعلاه كما ساهم في بناء القدرات و في التحسين للنظم للروتينية للإبلاغ عن السلّ وتسجيله وذلك عن طريق الإشراف الداعم المنتظم. كما أنه يبدو التدخلات المجتمعية الى جانب استراتيجية العلاج القصير الأجل الخاضع للإشراف المباشر DOTS strategy، لتمثّل المقاربة الأكثر فعالية في هذا الإطار. بيد أن هناك حاجة لمزيد من البحوث لدراسة مختلف نماذج متكاملة للتدخلات المجتمعية وذلك من أجل الفعالية النسبية لهذه القضية.

Translated from English version into Arabic by Thalia Rahme, through

TRANSLATORS WITHOUT BORDERS

结核病防治的社区干预

Ahmed Arshad, Rehana A Salam, Zohra S Lassi, Jai K Das, Imama Naqvi, Zulfiqar A Bhutta

摘要

2012 年，估计有 860 万人罹患结核病，130 万人死于该病。当前，结核病与 HIV 一起复燃，使其预防与管理面临更大的挑战。本文旨在评价社区干预对结核病防治的有效性。41 个相关研究表明，社区干预在结核病病例发现中具有显著作用（RR: 3.1, 95% CI: 2.92, 3.28），而仅有 1 项研究表明其在结核发病率方面无显著作用。在治疗活动性结核病时，社区干预可提高治疗成功率（RR: 1.09, 95% CI: 1.07, 1.11），且其中的 1 项研究表明干预后的复发率明显下降（RR 0.26, 95% CI: 0.18, 0.39）。这些结果适用于不同的研究设计和实施方案。定性综合分析显示，通过社区卫生工作者开展结核病的社区治疗不仅能提高访问和服务利用水平，还能促进能力建设，并通过定期的支持性监管改进结核病的登记和报告系统。社区干预联合短期直接观察治疗方案是最有效的措施。但是，仍需要更多的研究来评估各种社区干预实施案例的有效性。

Translated from English version into Chinese by Yin Jian-hai, edited by Yang Pin, through

CHINESE CENTER FOR DISEASE CONTROL AND PREVENTION
NATIONAL INSTITUTE FOR PARASITIC DISEASES

Interventions Communautaires ou locales pour la Prévention et le Contrôle de la Tuberculose

Ahmed Arshad, Rehana A Salam, Zohra S Lassi, Jai K Das, Imama Naqvi, Zulfiqar A Bhutta

RESUMÉ:
Près de 8.6 millions de personnes auraient contracté la tuberculose en 2012 et 1.3 en seraient décédés. La maladie associée au virus d'immunodéficience humaine (VIH) connait récemment une forte résurgence, ce qui rend la prévention et la gestion de la Tuberculose une tâche encore plus ardue et difficile. Ce document vise à évaluer l'efficacité des interventions à base communautaire ou locale pour la prévention et le traitement de la Tuberculose. Les conclusions tirées de 41 recherches sur l'efficacité des interventions communautaires laissent entendre que généralement ce genre d'actions dans le contexte de la prévention et du dépistage de cas, auraient un impact considérable sur la détection de la tuberculose (RR: 3.1, 95% CI: 2.92, 3.28), alors qu'une seule étude aurait révélé une incidence mineure de la tuberculose. Les interventions locales pour traiter les patients souffrant de tuberculose active ont montré une amélioration globale dans les taux de réussite des traitements (RR: 1.09, 95% CI: 1.07, 1.11) et les évidences d'une seule enquête suggèrent une réduction significative du taux de récurrence ou de rechute (RR 0.26, 95% CI: 0.18, 0.39). Les résultats tirés étaient homogènes et cohérents pour plusieurs modèles d'études et mécanismes d'exécution. La synthèse qualitative, elle, semble indiquer qu'un traitement de la Tuberculose par le biais d'interventions communautaires exécutées par des agents communautaires de la santé (ACS) nettement amélioré l'accès au dit service et son utilisation; mais aurait également contribué au renforcement des capacités et développé les système d'enregistrement et de déclaration systématiques de la Tuberculose et ce grâce à une supervision attentive et régulière. Les interventions à bases communautaires associées à la stratégie DOTS (traitement de brève durée sous surveillance directe), semblent constituer l'approche la plus efficace. Il est toutefois impératif d'entreprendre plus d'études pour évaluer les divers modèles communautaires intégrés et ce en vue d'une efficacité relative.

Translated from English version into French by Thalia Rahme, through

Территориальные меры, направленные на профилактику и контроль над туберкулезом

Ахмед Аршад, Рехана А Салам, Зохра С Ласси, Джай К Дас, Имама Накви, Зульфикар А Бхутта (Ahmed Arshad, Rehana A Salam, Zohra S Lassi, Jai K Das, Imama Naqvi, Zulfiqar A Bhutta)

ВЫДЕРЖКА:

В 2012 году у порядка 8,6 миллионов человек был обнаружен туберкулез, и более 1,3 миллиона человек скончались в результате этого заболевания. Ввиду недавнего возрождения заболевания наряду с вирусом иммунодефицита человека (ВИЧ), профилактика и лечение туберкулеза становится все более важной задачей. Целью этого документа является оценка эффективности предпринимаемых территориальных мер по профилактике и лечению туберкулеза. Данные из 41 исследования эффективности применяемых территориальных мер дают основание предполагать, что в целом применение территориальных мер для профилактики и выявления случаев заболевания оказало значительное влияние на обнаружение туберкулеза (RR: 3.1, 95% CI: 2.92, 3.28), в то время как влияние на распространение туберкулеза, в соответствии с одним исследованием, оказалось незначительным. Применение территориальных мер при лечении пациентов с активной формой туберкулеза позволило в целом улучшить показатели эффективности лечения (RR: 1.09, 95% CI: 1.07, 1.11) и, согласно данным одного исследования, частота рецидивов значительно снизилась (RR 0.26, 95% CI: 0.18, 0.39). Сходные результаты были в различных планах клинических исследований и при различных механизмах применения. Качественное обобщение предполагает, что территориальная доставка лекарств от туберкулеза через CHW не только улучшила доступ к медицинским ресурсам и повысила эффективность их использования, но также способствовала созданию потенциала и совершенствованию стандартных систем ведения учета и представления отчетности по случаям заболеваемости туберкулезом благодаря регулярному вспомогательному контролю.

Территориальные меры вместе с ДОТС-стратегией представляются наиболее эффективным подходом. Однако, необходимо проводить дополнительные исследования, чтобы оценить относительную эффективность комплексных территориальных моделей применения.

Translated from English version into Russian by tatiana_com, through

TRANSLATORS
WITHOUT BORDERS

Intervenciones basadas en la comunidad para la prevención y el control de la Tuberculosis

Ahmed Arshad, Rehana A Salam, Zohra S Lassi, Jai K Das, Imama Naqvi, Zulfiqar A Bhutta

ABSTRACTO:

En 2012, se estima que 8,6 millones de personas desarrollaron TB y 1,3 millón murió de la enfermedad. Con su reciente resurgimiento con el virus de inmunodeficiencia humana (VIH), la prevención y el manejo de la TB se ha vuelto más desafiante. Este documento tiene por intención evaluar la efectividad de las intervenciones basadas en la comunidad para la prevención y el tratamiento de la TB. Hallazgos de 41 estudios sobre la efectividad de la IBC sugieren que, en general, la IBC para la prevención de TB y detección de casos presentó un significativo impacto sobre la detección de la TB (RR: 3,1, 95% CI: 2,92, 3,28) mientras que hubo un impacto no significativo sobre la incidencia de la TB a partir de un único estudio. La IBC para tratar a pacientes con TB activa mostró una mejora general en las tasas de éxito del tratamiento (RR: 1,09, 95% CI: 1,07, 1,11) y evidencia de un único estudio sugiere una significativa reducción en la tasa de recaída (RR 0,26, 95% CI: 0,18, 0,39). Los resultados fueron consistentes para los diferentes diseños de estudio y mecanismos de entrega. La síntesis cualitativa sugiere que la entrega de tratamiento de la TB basado en la comunidad a través de Trabajadores de la Salud Comunitaria no sólo mejoró el acceso y la utilización del servicio sino que también contribuyó a la construcción de capacidad y mejoró los sistemas de reporte y registro de la TB de rutina a través de la supervisión de apoyo regular. Las intervenciones basadas en la comunidad junto con la estrategia DOTS parecen ser el enfoque más efectivo. Sin embargo, se necesitan más estudios para evaluar diferentes modelos de entrega integrada basada en la comunidad para una relativa efectividad.

Translated from English version into Spanish by Denise Tarud, through

TRANSLATORS
WITHOUT BORDERS
